# Occurrence of oral habits among preschool children with Autism Spectrum Disorder

**DOI:** 10.12669/pjms.335.13554

**Published:** 2017

**Authors:** Fares S. Al-Sehaibany

**Affiliations:** 1 Fares S. Al-Sehaibany, BDS, DMSc, Associate Professor, Department of Pediatric Dentistry and Orthodontics, College of Dentistry, King Saud University, Riyadh, Saudi Arabia

**Keywords:** Autism spectrum disorder, Oral habits, Preschool children

## Abstract

**Objective:**

To determine occurrence of oral habits among Saudi preschool children with autism spectrum disorder (ASD) and compare it with healthy preschool children.

**Methods:**

This study was conducted over a 14-months period in Riyadh, Saudi Arabia. The sample consisted of two groups; a study group (SG) of 150 ASD children, and a control group (CG) of age- and gender-matched 150 healthy children. The parents of the children in both the groups were administered a questionnaire that included questions about the children’s demographic information and previous or persistent oral habits.

**Results:**

The prevalence of oral habits was higher (87.3%) among the SG children as compared to CG children (49.3%). The most prevalent oral habit among the SG was bruxism (n = 82; 54.7%), followed by object biting (n = 67; 44.7%) and mouth breathing (n = 40; 26.7 %). Among the CG; the most prevalent oral habit was mouth breathing (n = 40; 26.7%) followed by nail biting (n=18; 12%) and object biting (n = 7; 4.7%). The prevalence of bruxism, object biting, thumb sucking and tongue biting was significantly (p<0.05) higher in the SG than the CG.

**Conclusions:**

The prevalence of oral habits was higher in the ASD group children than the healthy children.

## INTRODUCTION

Autism or autism spectrum disorder (ASD) is a group of neurodevelopmental disorders that may affect children at an early age.^1^ In Saudi Arabia, the reported prevalence of ASD was 18 per 10,000 live births in 2009.^2^

In addition to self-injurious behaviors such as hitting with bare hands, banging their heads on walls and furniture, and pricking or pinching;^3^ oral habits including bruxism, tongue thrusting, lip biting, and pica (eating objects and substances such as gravel, or pens) have been reported among children with ASD.^4^ These habits may contribute to significant dental problems such as soft tissue injury, tooth loss, tooth wear, increased overjet, anterior open bite, and posterior crossbite.^5^ Furthermore, these habits are associated with skeletal and dentoalveolar deformation. The severity of deformation is related to the frequency and duration of the habit, and should be evaluated by pediatric dentists.^6^ An assessment of the prevalence of various oral habits among children with ASD and comparison non-ASD children would provide the necessary information regarding the habits in these groups and the problems associated with the oral habits. Studies investigating the prevalence of oral habits among children with ASD are relatively rare. Therefore, the aim of the present study was to determine occurrence of oral habits among Saudi preschool children with ASD and compare it with healthy preschool children.

## METHODS

The study protocol and consent form were approved by the Research and Ethical Committee of Human Studies at the College of Dentistry Research Center (PR 0024), in King Saud University, Riyadh, Saudi Arabia. The sample consisted of 150 SG children (3-6 years old) and 150 age- and gender-matched CG children. The SG was recruited from three ASD centers randomly selected from a list of ASD centers obtained from the Saudi Ministry of Education. Three kindergartens were also randomly selected from a list of kindergartens provided by the ministry. A letter explaining the objectives of the study and informed consent forms were sent to the parents of the selected children through the kindergarten principals. Parents who agreed to participate in the study received a self-administered questionnaire for completion.

The study was conducted over a 14-month period between September 2014 and October 2015. The questionnaire was derived from the oral habits guidelines of the American Academy of Pediatric Dentistry^6^ and was translated from English to Arabic by a certified bilingual translator. The questionnaire asked about the child’s gender, date of birth, medical history, previous history of orthodontic treatment, and previous or current oral habits. A test-retest was performed to verify the consistency and reliability of the questionnairein parents of 15 SG and 15 CG children not participating in the main study.

The data obtained from the questionnaires were manually entered into the computer using Statistical Package for the Social Sciences software package (IBM, SPSS version 20, Chicago, IL, USA) and analyzed using a significance level of P<0.05. The statistical analyses included frequency distribution, cross-tabulation, Fisher’s exact test, and Pearson’s Chi-square test to compare the two groups.

## RESULTS

**Fig.1:**
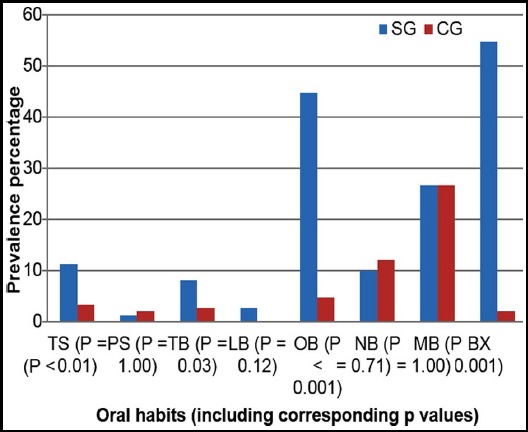
The prevalence of oral habits in the SG and CG. SG: study group, CG: control group, TS: thumb sucking, PS: pacifier sucking, TB: tongue biting, LB: lip biting, OB: object biting, NB: nail biting, MB: mouth breathing, BX: bruxism.

The age of the children in each group ranged between 3-6 years with a mean age of 4.7 ± 0.8 years for SG and 4.4 ± 0.6 years for CG. In each group the number of males (n=109; 73%) was significantly (p<0.001) higher than females (n=41; 27%), the male:female ratio was 2.7:1. Oral habits were prevalent among 131 (87.3%) of the SG children and 74 (49.3%) of the CG children. There were no gender differences in both the groups, so combined data are presented. The prevalence of oral habits for the SG and CG children is shown in Fig.1. The most prevalent oral habit among the SG was bruxism (54.7%), followed by object biting (44.7%) and mouth breathing (26.7 %). Among the CG, the most prevalent oral habits were mouth breathing (26.7%) followed by nail biting (12%) and object biting (4.7%). The prevalence of bruxism, object biting, thumb sucking and tongue biting was significantly (p<0.05) higher in the SG than the CG.

The duration (hours per day) of various oral habits in the SG and CG is shown in Table-I. Half of the children (50%) in the SG showed a bruxism duration of more than one hour, while no child in the CG showed a bruxism duration of more than one hour. Similarly, higher number of children in the SG showed reported object biting duration (n=29) and tongue biting duration (n = 10) of more than one hour daily than the CG (n=6 and 0, respectively).

**Table-I:** The duration per dayof oral habits in the SG and CG.

*Oral Habit*	*Group*	*Duration*				*P value*

		*≤ 1 hr. n (%)*	*>1 - 3 hrs. n (%)*	*> 3 - 6 hrs. n (%)*	*> 6 hrs. n (%)*	
TS	SG	11 (64.6)	2 (11.8)	2 (11.8)	2 (11.8)	< 0.001
	CG	0 (0.0)	4 (80.0)	0 (0.0)	1 (20.0)	
PS	SG	0 (0.0)	2 (100.0)	0 (0.0)	0 (0.0)	< 0.001
	CG	0 (0.0)	3 (100.0)	0 (0.0)	0 (0.0)	
TB	SG	2 (16.7)	1 (8.3)	8 (66.7)	1 (8.3)	< 0.001
	CG	4 (100.0)	0 (0.0)	0 (0.0)	0 (0.0)	
LB	SG	4 (100.0)	0 (0.0)	0 (0.0)	0 (0.0)	< 0.001
	CG	0 (0.0)	0 (0.0)	0 (0.0)	0 (0.0)	
OB	SG	38 (56.7)	27 (40.3)	2 (3.0)	0 (0.0)	< 0.001
	CG	1 (14.3)	6 (85.7)	0 (0.0)	0 (0.0)	
NB	SG	14 (93.3)	1 (6.7)	0 (0.0)	0 (0.0)	< 0.001
	CG	0 (0.0)	17 (94.4)	1 (5.6)	0 (0.0)	
MB	SG	1 (2.5)	26 (65.0)	3 (7.5)	10 (25.0)	< 0.001
	CG	3 (7.5)	30 (75.0)	2 (5.0)	5 (12.5)	
BX	SG	41 (50.0)	26 (31.7)	12 (14.6)	3 (3.7)	< 0.001
	CG	3 (100.0)	0 (0.0)	0 (0.0)	0 (0.0)	

SG: study group, CG: control group, TS: thumb sucking, PS: pacifier sucking, TB: tongue biting, LB: lip biting, OB: object biting, NB: nail biting, MB: mouth breathing, BX: bruxism.

The pattern of oral habits (while asleep, awake or both) for the SG and CG children are shown in Table-II, A majority in the SG reported bruxism while awake (69.5%). Similarly, majority of the children in the SG reported object biting while awake (97%). Whereas all of the CG children with bruxism and object biting reported performing the habits while awake.

**Table-II:** The pattern of oral habits in the SG and CG.

*Oral Habit*	*Group*	*Pattern*			*P value*

		*While asleep n (%)*	*While awake n (%)*	*Both n (%)*	
TS	SG	2 (11.8)	11 (64.6)	4 (23.6)	< 0.001
	CG	0 (0.0)	0 (0.0)	5 (100.0)	
PS	SG	0 (0.0)	0 (0.0)	2 (100.0)	0.480
	CG	2 (66.7)	0 (0.0)	1 (33.3)	
TB	SG	1 (8.3)	1 (8.3)	10 (83.4)	< 0.001
	CG	3 (75.0)	1 (25.0)	0 (0.0)	
LB	SG	1 (25.0)	0 (0.0)	3 (75.0)	< 0.001
	CG	0 (0.0)	0 (0.0)	0 (0.0)	
OB	SG	0 (0.0)	65 (97.0)	2 (3.0)	< 0.001
	CG	0 (0.0)	7 (100.0)	0 (0.0)	
NB	SG	0 (0.0)	15 (100.0)	0 (0.0)	< 0.001
	CG	0 (0.0)	18 (100.0)	0 (0.0)	
MB	SG	25 (62.5)	3 (7.5)	12 (30.0)	< 0.001
	CG	8 (20.0)	2 (5.0)	30 (75.0)	
BX	SG	9 (11.0)	57 (69.5)	16 (19.5)	< 0.001
	CG	0 (0.0)	3 (100.0)	0 (0.0)	

SG: study group, CG: control group, TS: thumb sucking, PS: pacifier sucking, TB: tongue biting, LB: lip biting, OB: object biting, NB: nail biting, MB: mouth breathing, BX: bruxism.

The time length (in years) of the oral habits in the SG and CG children is shown in Table-III. A majority in the SG had bruxism for more than one year (82.9%). Similarly, majority of the children in the SG had the habits of tongue biting (100%), object biting (64.6%) and thumb sucking (64.6%) for more than two years. The time length of all the oral habits in the majority of CG children, was more than one year.

**Table-III:** The time length (in years) of oral habits in the SG and CG.

*Oral Habit*	*Group*	*Time Length*			*P value*

		*< 1 yr. n (%)*	*1 - 2 yrs. n (%)*	*> 2 yrs. n (%)*	
TS	SG	3 (17.7)	3 (17.7)	11 (64.6)	< 0.001
	CG	0 (0.0)	5 (100.0)	0 (0.0)	
PS	SG	0 (0.0)	2 (100.0)	0 (0.0)	0.480
	CG	1 (33.3)	2 (66.7)	0 (0.0)	
TB	SG	0 (0.0)	0 (0.0)	12 (100.0)	< 0.001
	CG	1 (25.0)	2 (50.0)	1 (25.0)	
LB	SG	1 (25.0)	1 (25.0)	2 (50.0)	< 0.001
	CG	0 (0.0)	0 (0.0)	0 (0.0)	
OB	SG	4 (5.9)	20 (29.5)	43 (64.6)	< 0.001
	CG	2 (28.6)	5 (71.4)	0 (0.0)	
NB	SG	2 (13.3)	8 (53.4)	5 (33.3)	< 0.001
	CG	0 (0.0)	14 (77.8)	4 (22.2)	
MB	SG	2 (5.0)	23 (57.5)	15 (37.5)	< 0.001
	CG	3 (7.5)	21 (52.5)	16 (40.0)	
BX	SG	14 (17.1)	46 (56.1)	22 (26.8)	< 0.001
	CG	0 (0.0)	0 (0.0)	3 (100.0)	

SG: study group, CG: control group, TS: thumb sucking, PS: pacifier sucking, TB: tongue biting, LB: lip biting, OB: object biting, NB: nail biting, MB: mouth breathing, BX: bruxism.

## DISCUSSION

The present study has provided important baseline information on the occurrence of oral habits in a group of Saudi preschool children with ASD. Development of dental occlusion is strongly influenced by environmental factors such as oral habits. Early diagnosis and successful treatment of oral habits is pivotal in the development of occlusal harmony and function.^7^ Therefore, intervention leading to oral habits cessation should be initiated as early as possible.

There are several reasons why the children with ASD receive limited oral health care including preventive care. These reasons include lack of knowledge and experience regarding ASD among dental professionals and a low priority for preventive oral health care on part of the parents.^8^,^9^ A better understanding of the behavioral effects of ASD and existing oral habits may help dental practitioners deliver preventive oral health care empathetically and appropriately.^10^

The present study found that bruxism was the most prevalent oral habit in the SG. Furthermore, the duration and time length of bruxism were higher in the SG. Studies have reported that bruxism is a common habit among children with ASD.^11^ Bruxism in children with special healthcare needs may result in excessive wear of dentition, and temporomandibular joint (TMJ) pain.^12^ The use of splints or behavioral modification techniques to treat bruxism may be limited in children with ASD due to their poor intellectual skills and communication difficulties.^13^ Intraoral appliances, such as splints, may become an aspiration hazard for children with ASD.^12^ Therefore, pharmacological treatment has been recommended ranging from injection of botulinum toxin to the use of central nervous system medication.^12^,^14^

In the present study, significant differences in the prevalence of bruxism, object biting, thumb sucking, and tongue biting was observed between the SG and CG. Higher prevalence of bruxism, object biting and thumb sucking habits among the SG may lead to TMJ pain and malocclusion. The resulting TMJ pain and malocclusion require specialists’ consultation and treatment, resulting in high financial expenses for parents of the children with ASD. The higher prevalence of tongue and object biting in the SG may also be attributed to the altered pain tolerance or expression that has been reported in children with ASD.^15^

Pediatric dentists can expect to face the challenge of providing preventive dental care to an increasing number of children with ASD.^16^ A reduced oral health-related quality of life as perceived by the parents of Saudi children with ASD has been documented.^17^ This could be attributed to fact that the parents are overwhelmed by the medical and behavioral problems of their ASD children, resulting in lower priority to dental health.^18^ Therefore, the provision of preventive dental care and increasing dental health knowledge of the parents of ASD children is of vital importance.

## CONCLUSIONS

The most prevalent oral habit in the SG was bruxism, whereas the most prevalent oral habit in the CG was mouth breathing. The prevalence of bruxism, object biting, thumbs sucking and tongue biting was higher in the SG than in the CG.
